# Collectanea Medica: Consisting of Anecdotes, Facts, Extracts, Illustrations, &c.

**Published:** 1825-03

**Authors:** 


### COLLECTANEA MEDICA:

CONSISTING OF

### ANECDOTES, FACTS, EXTRACTS, ILLUSTRATIONS, &c.

*Relating to the History or the Art of Medicine, and the Collateral Sciences.*

Floriferus, ut apes, in saltibus omnia libant,

Omnia nos, itidem, depascimur aurea dicta.

---

**Art. I.**—*Extracts from "An Analysis of Medical Evidence, &c."* By John Gordon Smith, M.D.

### *On the most advisable Manner of giving Evidence.*

To this chapter one might fancy all the lame portion of the community hastening, regardless of what precedes or what follows, in the hope that a miraculous healing will attend the dip. But, alas! my friends, this is not a pool for the cure of your malady. If such be your views of your own case, many of you must be too far gone to hope for relief; and, if so, all you can rely upon for comfort is the chance (that has happened to some) of being able to avoid exciting causes. There is no nearer road to the realization of your wishes, than to begin at the beginning of that knowledge which alone can stand you in stead, and which, as it is not the object of the whole volume to impart it, you ought not to expect from any individual chapter. If this attempt will at all aid my brethren of the medical profession, it must arise from consideration of the whole; for the distinctions I have aimed at are merely for the convenience of the reader, and not with any view to a symmetrical arrangement of the work. I attach little importance to them myself, and consider a perusal of the whole as indispensable to him who really desires to profit by the subject.

The oath administered to witnesses in the English courts binds them *to speak the whole truth, and nothing but the truth, touching the matter in question.* Explicit as this formula may seem, and convenient as it may often be found on the part of a lawyer, for the purpose of frightening a timid witness,—plain as it might appear to make the duty of the witness, even in his own estimation,—it seems applicable chiefly to matters of *fact,* and hardly consistent with that which should be the prescriptive rule as to matters of *opinion.* We have heard of anomalies in the sense of vision, causing the person in whom they existed to mistake one colour for another. Should, therefore, a man, through such vitiation of his perceptive powers, swear that a red coat was a black one, we could not receive his statement as evidence, although the honest expression of his belief. Deviations equally great take place—more frequently, perhaps, than we are aware of, in matters of opinion; and yet those who utter them may be as little chargeable with wilful or conscious error, as the unfortunate being who labours under such a morbid affection of the optic nerve as that alluded to.

As to matters of opinion, therefore, it is natural to ask, in what way should the whole truth be displayed? in what manner is it to be communicated on points of science?

Some have affected to lay down one rule, while others have pretended that the opposite would be more suitable; as if it were possible to furnish any rules for behaviour, under circumstances that vary like the aspect of the clouds, and cannot be fixed as a basis for instruction. But, although we give up the pretence to talk in a didactic tone, we are fully entitled to rely upon a general principle, the knowledge and applicability of which must, after all, be acquired rather through an innate sense of propriety, than derived from any formal attempt at instruction.

I understand that certain teachers of other branches (for the lesson could hardly be inculcated by one who knows this subject,) desire their pupils, when called into a court of justice, to be niggard of their knowledge, and to utter nought beyond a bare answer to the question. I remember being in company with a witling, when he bestowed a farthing upon a beggar, accompanying the gift with a very grave admonition *not to lay it all out at once:* and I hesitate not to add, that the injunction, though it may be appropriate as far as the teacher is concerned, is mere mockery of the pupil, unconscious, as he must be, of the nature of the occasion to which his inconsiderate preceptor refers. I cannot imagine, viewing the caution in the abstract, how it could be reducible to any purpose of practical utility. Could we suppose both interrogator and respondent to be equally and consummately learned on the point at issue, the one would, in all probability, ask such questions only as the other would be most readily able to answer; but, even in such a state of things (unhoped-for as it may be), the concise form of reply might not suit the judges, jury, cross-examiners, &c.; and the advice must be founded (if founded in any thing but utter ignorance of the forum,) upon a supposition that we are only to be asked questions relating to matters of fact,—such as, what the hour was when we received a coroner's summons? or how long it took us to travel to the inquest? But are we not generally called upon because the court knows it cannot, of itself, rely upon eliciting the truth in matters of science and of opinion, that demand the undivided attention of men, supposed to be equal as to preliminary education, preparation, and acquirements, with the members of the judiciary orders themselves, and who have devoted their lives to the exclusive study of these matters? It is absurd to expect that questions can be so framed, that a simple *yea* or *nay* should answer them. I anticipate, for my own part, no small annoyance, perhaps occasional censure, and it may be even rejection as a witness, from a determination not to be cramped by the narrow views of an advocate, in giving professional testimony; but, being sufficiently forewarned as to the impropriety of hurrying through my business as a witness, I should be loth to yield to an overbearing counsel an inglorious triumph over the importance of medical science, or the interests and respectability of medical men, as far as I may be capable of perceiving that these are endangered.

Once more, with regard to this very inept injunction. Can its authors guarantee their uninitiated and unsuspecting pupils, that such meagre replies as they are considered to make, (supposing that the advice conveyed with it the power of Lacedæmonian concision,) will prevent the advocate from repeating or varying his questions, or from amplifying the course of his examination? Really, the thing is too absurd to be dwelt upon.

Others again recommend that we should say every thing that occurs to us relative to the point at issue; and, of the two, such an injunction is the more reprehensible. In many cases, this is advising to the performance of impossibilities. There are some who might, by such a course, save the time of the court, and convey, in the most convincing manner, the clearest views of the truth; but, for one who could accomplish this, there are many who might in vain attempt it. Supposing, however, that in all cases it might be accomplished with equal advantage, it is not always permitted; and many instances are to be found where the attempt has been prevented. It would not always suit the purpose of the lawyer, who, knowing what it would be desirable to elicit, and apprehensive, perhaps, of something coming out that his object requires should be concealed, will studiously put his questions in such a way as shall mould the intelligence of the witness to the purpose. Among the few, but very apposite, observations on evidence, with which we are favoured by the authors of a work that at present deservedly forms the standard of our medico-legal knowledge, is an overwhelming of the court by *a flow of garrulity.* Upon this it may not be impertinent to remark, that a witness may gallop off in this way, and perhaps proceed to some distance—*sed cui bono?* "Stay a little, and we shall have done the sooner," is reported to have been said on some such occasion by a judge; and the witness who plays such a prank will have to begin again, and go over the same ground with deliberation. It is indispensably necessary that the evidence should be written by the judge, if he has to recapitulate it to the jury; and such is the practice: consequently, the statements of the witness, whether elicited piece-meal by a chain of interrogations, or given in the form of a connected narrative, must be uttered with that slowness and precision, which partake rather of the nature of dictating to an amanuensis, than an attempt to outstrip a stenographer. This being the case, I am at a loss as to dissuading against a practice that can hardly be contemplated. One thing appears very manifest, viz. that a witness who might be thoughtless enough to indulge in such a style of address, and might at the same time be permitted to proceed a certain length, would be very likely to lay himself open to a severe cross-examination, by furnishing hints to the counsel, that a more cautious and considerate person would not be so likely to do.

"We recommend the witness," says Messrs. Paris and Fonblanque, "to steer a middle course: first, answering patiently, distinctly, and tersely, the questions put by the counsel on both sides, the court, and the jury; and if none of these elicit the whole truth, and any material point remains to be disclosed, the presiding judge will always admit, and gratefully receive, the additions or explanations which may be necessary to the ends of justice." This recommendation is dictated by correct views of the matter, and is precisely the caution I should think it my duty to give. But I feel it necessary to be rather more particular, and to explain more fully to my brethren some things that are not laid down with sufficient clearness in the foregoing passage.

Let me then follow up the caution of these gentlemen as to the article of *patience.* Provide yourselves with a large stock, and "do not lay it all out at once." If you go forward to an occasion of witness-bearing, whose interests are of magnitude, eager to get through the business, and pass to some one more agreeable or more profitable, you will be disturbed in the discharge of your duty, your usefulness will probably be destroyed, and you may then save as much character from the wreck as you can. And let me present the same lesson in a form somewhat more familiar—*Keep your temper!* There, the lawyers themselves acknowledge, lies the great secret. Be not accessory to making your examination a *cross* one, in the sense alluded to in the sequel. You are cautioned to speak "distinctly and tersely:" and this quotation I shall elucidate by another. "The important duty which the medical practitioner has to perform, when he delivers his testimony before a court of justice, should be closely defined, conscientiously felt, and thoroughly understood: his opinion ought to be conveyed in a perspicuous manner." Thus, to patient consideration of the merits of the question that may be put, or of the case you may be called upon to describe, you must add attention to fit and appropriate language. In private life, when a party does not comprehend what is said by another, the cause may be ascribable to deficiency of apprehension on the one side, as justly as to incapacity for imparting on the other; but surely, where a whole court is unable to perceive the meaning of a witness, it is more likely that *he* shall be a stupid fellow, than that an assembly of lawyers shall be deficient in the acuteness that proverbially belongs to them. But I should, in my own case, be ambitious to go a step beyond this. I should like not only to convey my meaning in terms that they might not be at liberty to misinterpret, but such as they could receive in no sense but the proper one. We read of the naked truth, and the nearer this familiar standard of simplicity we approach in our descriptions of it, we shall do the more good, and suffer the less annoyance.

Whether the advice of my judicious fellow labourers in this branch of public interests be approved of or not,—whether our readers decide for "costive retention," or a flow of garrulity, there is one very important point which they will do well to look to—*Let them think!*

Exceptions as to general facts, or established practices relating to the point at issue, claim very marked notice in this place. There is a wide difference between founding an opinion, or giving testimony upon rare deviations from the course of nature or of events, and omitting to notice exceptions that belong as strictly to the rule as the circumstances that constitute it. A scientific witness would be truant to his own character if, when publicly called upon to speak of his knowledge, he gave an imperfect or an erroneous statement, which it might fall to the share of others to correct; and it does appear to me that it would be little more in his favour if he corrected himself upon compulsion. The advocate who has to conduct the indirect examination, and he also who might re-examine, through whom alone can the witness have the opportunity of making such corrections, may, either from being unacquainted with the whole bearings of the subject, or from other causes, omit to furnish him with it. The report goes out to the world, and the testimony of the medical man runs the gauntlet of all those who may be able or disposed to display its defects, and more serious vices. An example, figurative of what is here alluded to, took place under circumstances of unusual interest, not long ago; and a lesson was drawn in one of the professional journals, which I think may be appropriately quoted.—Speaking of one of the witnesses, it is observed, "This gentleman seems to have been truly unfortunate in the grounds of his opinion. It would appear as if he had formed it from the details afforded by a servant,—and that a discarded one. *In his cross-examination, also, he admits exceptions enough to eat up his evidence in chief.* It is with no small regret that we find ourselves compelled to animadvert upon the *testimoniary* deportment of men of eminence; but it has been the fate of the most eminent, occasionally, to have given too much scope for animadversion on similar occasions: and we seize the occasion to say to our younger brethren, that to us, who are in the habit of looking at medical practitioners in the situation of witnesses with a scrutinising eye, they appear in a disadvantageous light, when they allow most important and most manifest parts of the truth to be wrung from them by a cross-examination."

It is surely unnecessary to say any thing more about speaking dubiously. It would be a decided dereliction of duty to speak thus, when we can do it positively; but it may be often right to speak even in that manner. Leaving the fact in doubt, is not leaving the accused in danger; but, the caution actually required here, it is hardly possible to convey in concise language. In a more circumlocutory form, it appears to me that the reluctance of these persons relates to the risk that may follow, should they leave in doubt any point upon which they may be required to speak, and that they consider it their duty to speak positively, or plead total ignorance. Now this is not fair, either towards science, or towards the persons interested in what is going on; and I should prefer giving even an undecided opinion, where the subject is not known to have an accredited form: otherwise, its real importance may be under-rated; or it may be unjustly considered to be a visionary hypothesis, when it merely defies satisfactory explanation.

In consequence of our being sworn to disclose the whole truth, we may be called upon to reveal secrets confided to us in professional confidence. This involves a very delicate consideration, and one that I apprehend is but imperfectly understood. "Barristers and attorneys, to whom facts are related professionally during a cause, or in contemplation of it, are neither obliged nor permitted, though they should so far forget their duty as to be willing to do so, to disclose the facts so divulged, during the pendency of that cause, or at any future time."—

"This rule of professional secresy extends only to the case of facts stated to a legal practitioner, for the purpose of enabling him to conduct a cause; and, therefore, a confession to a clergyman or priest, for the purpose of easing the culprit's conscience, the statement of a man to his private friend, or of a patient to his physician, are not within the protection of the law. We should certainly think that the friend or the physician who voluntarily violated the confidence reposed in him, acted dishonourably; but he cannot withhold the fact, if called upon in a court of justice."

I am by no means able to venture upon a question of such magnitude, and hardly know how to choose language for the conveyance even of a doubt for others to satisfy. That the practice of the courts in Great Britain is in strict accordance with the foregoing view of the matter, every one well knows; and it may be equally certain that they possess the *right*, as well as the *power*, of compelling witnesses to such disclosures; but to my apprehension it is not equally clear. A precedent is, in law, a mighty authority; and I am quite satisfied that a point which has been so often and so uniformly ruled, will never be ruled otherwise in the courts of Westminster Hall: I am also well aware that to *law*, and rules of court, we must yield, or the administration of justice would be impeded. But, although satisfied on those points, I am not contented that we should be placed beyond the pale of those, to whose private and confidential dealings with their fellow citizens such respect is shown. I will not go at large into the question, my design being merely to draw the notice of my brethren to the circumstance, and to put them upon their guard as far as possible; yet will I say that circumstances may occur, in which a man of a delicate and honourable mind, being the depositary of certain things communicated to him, either under the seal of professional or private confidence, (for the distinction is not to be entertained here,) would endure much ere he would reveal. But such a situation is peculiarly distressing, because, on the supposition that resistance might be maintained, he not merely exposes himself to personal suffering, but incurs the charge of disregard to the interests of justice, and dereliction of duty to his country: in other words, he will be considered as allowing the private claims of an individual to set aside those of the public weal. Let it be distinctly understood, before I go farther, that I am not alluding to the case of the priest and a culprit's conscience, but to matters (it may be) of the last importance to the character of individuals and the peace of families, arising out of circumstances of a purely private nature, and in no way relating to affairs of state or municipal interest. It will at once strike the manly mind that, in regard to females, we might be called upon to reveal that, of which the promulgation would, to them, be worse than death itself.

All writers on this subject are not of the same opinion. In "Medical Jurisprudence," it is noticed in a cursory manner, and we are informed (rather, I suppose, by Mr. Fonblanque than by Dr. Paris,) that, "when the ends of justice absolutely require the disclosure, *there is no doubt* that the medical witness is not only bound, but compellable, to give evidence, ever bearing in mind that the examination should not be carried further than may be relevant to the point in question: of this the court will judge, and protect the witness accordingly." This is unquestionably sound doctrine, as drawn from the practice of our courts; but as, in my very humble apprehension, the matter seems to be one remounting, in its original bearings, rather to a question of general right, than of the law of any particular state, I beg to quote, from a good French writer on Legal Medicine, a sentiment of rather an opposite tendency:—"The tribunals neither ought, nor have the power to exact from a physician the revelation of a secret confided to him in consideration of his office: at all events he may, and ought to refuse. Religion, probity, nay the rights of society, make this a law. Still more are we bound to secresy, when not compelled to disclose. Upon this point, casuists and jurisconsults are of one opinion."

Zacchius enumerates *loquacity* among the errors of physicians cognizable by law, and censures it as particularly deserving of punishment, when it leads to the disclosure of secrets: upon which he alludes to the clause binding to secresy, in the celebrated oath of Hippocrates, as an obstacle on the part of those who have taken it; and, in his famous casuistical way, first puts a case or two, and then leaves us the full force of the dilemma to contend with. I must add, that he distinguishes between judicial revelation and that which is not; "Nam in judicio tenetur omnino veritatem detegere;" yet even here he seems to be in the same predicament as myself, with regard to the original merits of the question; for he does no more than tell us, that to make such disclosures *is held to be* necessary.

Perhaps, the apparent freedom of these remarks may lead to further inquiries on the subject: in the mean time, and with a view to my own justification for having taken the liberty to express unwonted anxiety as to this point, I may repeat the grounds on which I think myself warranted in having done so. In the first place, lawyers are not only privileged in maintaining secresy, but are not permitted to violate it when inclined; and justice should, in my apprehension, respect private delicacy and reputation, as well as property,—more especially when the former are the more valuable in the estimation of the possessor; and, secondly, as I consider physicians to be virtually bound by the spirit, if not by the letter, of the Hippocratic oath, I am not satisfied that the strong and overbearing voice of authority would be adequate to release me from the misgivings of my own mind, after having revealed matters that I never should have known, but from an implied belief in the force of my professional obligations to be secret. In order, however, to discharge my duty properly to such of my brethren as may not view the matter in the same light, I must add that, in the great majority of cases where disclosures of the kind are called for, there may be no real breach of confidence, because society in general receives the authority of courts as paramount to all obstacles and private considerations; so that, in yielding to such authority, a professional man will be fully acquitted, even in the opinion of those who may be the sufferers. Of course, therefore, there is an implied exception in regard to such a call, and the as in the case of a Catholic priest.

practitioner may be less embarrassed by the scruples of those whom he may commit, than by his own.

But I cannot dismiss the subject without cautioning the young practitioner as to his mode of proceeding under such circumstances. To an advocate no such revelations are to be conceded, let him demand them ever so urgently; and I should hold that barrister personally amenable who would presume to ask me to disclose a secret, as a matter of course, merely upon his requisition. A *gentleman* will certainly hesitate as much in requiring, as another would in affording, such disclosures; and they are never to be made, but by express mandate from the bench. That being uttered, I can disapprove of no one's conduct in obeying. The expressed opinion of the judge will be a full indemnity for the witness, as far as all *extra-thoracic* dissatisfaction might exist: but I cannot be satisfied until I shall have expressed my regret, that, when the sacred barrier of private confidence must be thrown down, it is to be done in so public a manner. Surely, on such occasions, the idle and unconcerned at least might, with great propriety, be excluded, as well as on others that I need not specify.

### *The subject continued—more particularly with regard to the observance of Decorum.*

Let us then go to this duty without a humiliating apprehension, which will infallibly injure us. Let us leave bad temper and a suspicious disposition,—leave impatience, if these be the infirmities of our nature, at home. Let us carry with us our share of that commodity, so necessary on all occasions, viz. self-respect; and clothe ourselves in our best manners, and choicest suit of intellectual garniture. Let us maintain the spirit, and observe the deportment, that should exist between gentlemen; and if there should, by rare hazard, occur any cause for dissatisfaction, comport ourselves even then in the same capacity. I grant the occasion is one that brings inevitable uneasiness to many; but I cannot help thinking that, to a competent witness, it is very much of a bugbear in reality. I will admit, that now and then a disposition may be manifested to treat a witness unfairly, and that it is hard to say to what one may be provoked who allows this to take effect on his irritability; but the proper way of defence for him who may be assailed in this manner, will be an appeal to the court.

Of all things, we should be cautious as to trying the lawyer at his own weapons. It has been well represented by a medico-legal writer, whose own conduct under a most teazing and overbearing course of examination I shall have occasion to exemplify, that "the lawyer's object being the interest of his employer, for the fulfilment of his duty he is frequently compelled to resort to a severity of investigation which perplexes the theories, but more frequently kindles the irritable feelings, of the medical practitioner: however dexterous he may show himself in fencing with the advocate, he should be aware that his evidence ought to impress the judge, and be convincing to the jury."

The practical application of this may be considered very simple: at all events it must be left, in great measure, to the circumstances and particular taste of the individual concerned: and, with a single additional hint, I shall pass to another point, hoping that what has been adduced may have the effect of turning the mind into the proper channel for strengthening itself for the contest. I know some who are perfectly capable of trimming even a lawyer, and in a way that would be effectual enough. Such will not be offended with me, if I intreat them to reserve their *wit* for other occasions. If to them, personally, the indulgence of the impulse might not be dangerous, the example would be highly so; and I am persuaded that they will consult their own dignity and interest better by not treating lightly the serious business of a court of justice. In some the natural impulse is so powerful, as to render it impossible not to retort, when assailed, with that very dangerous weapon, and upon such it might be vain to enjoin absolute forbearance: I shall, therefore, restrain my exhortation to putting a check on the propensity, and to avoid any attempt to give what is termed a *set down*. If such an upshot supervenes as a matter of course, it may all be very well: but I shall conclude this chapter with an exemplification of the error against which this last caution is more particularly directed.

Not very long ago, an apothecary, who had previously been clerk to a barrister, was examined as a witness in the Court of King's Bench. One of the most eminent counsel of the present day was particularly desirous to ascertain from him how long he had changed his calling; and at length drew upon himself the following piece of intelligence:—"I began the study of medicine at a much earlier period of life than the late Lord Erskine did that of law; and he attained to far greater eminence in his profession than ever you will!" For this retort he was deservedly chastised by the judge, who, in recapitulating the evidence, very properly remarked, that, whatever that witness might have learned in his two professions, he had not learned manners; for the answer he had given to Mr. ——— was very impertinent.

